# Exploring the capabilities of monochromated electron energy loss spectroscopy in the infrared regime

**DOI:** 10.1038/s41598-018-23805-5

**Published:** 2018-04-04

**Authors:** Jordan A. Hachtel, Andrew R. Lupini, Juan Carlos Idrobo

**Affiliations:** 1 Center for Nanophase Materials Sciences, Oak Ridge National Laboratory, Oak Ridge, TN 37831 United States of America; 2 Materials Science and Technology Division, Oak Ridge National Laboratory, Oak Ridge, TN 37831 United States of America

## Abstract

Monochromated electron energy loss spectroscopy (EELS) is one of the leading techniques to study materials properties that correspond to low (<5 eV) energy losses (*i.e*. band-gaps, plasmons, and excitons) with nanoscale spatial resolution. Recently a new generation of monochromators have become available, opening regimes and unlocking excitations that were previously unobservable in the electron microscope. The capabilities of these new instruments are still being explored, and here we study the effect of monochromation on various aspects of EELS analysis in the infrared (<1 eV) regime. We investigate the effect of varying levels of monochromation on energy resolution, zero-loss peak (ZLP) tail reduction, ZLP tail shape, signal-to-noise-ratio, and spatial resolution. From these experiments, the new capabilities of monochromated EELS are shown to be highly promising for the future of localized spectroscopic analysis.

## Introduction

Electron energy-loss spectroscopy (EELS) is a technique commonly used within a scanning transmission electron microscope (STEM) to provide high-spatial-resolution spectroscopic data^1–3^. EELS measurements have two standard regimes: core loss (>50 eV), where compositional and electronic information can be obtained from the electron beam interaction with deep core states, and low loss (<50 eV) where optical information can be determined by examining excitations into low-lying states above the Fermi energy^4^. The precision of an energy loss measurement is limited by spread of the electron energies coming out of the field-emission electron gun. These spreads are typically small (<1 eV), and for many applications of EELS, such as core-loss elemental mapping and bulk plasmon measurements, they are tolerable. However, for excitations with narrow linewidths (e.g., surface plasmons or excitons) as well as fine-structure analysis, this spread can mask multiple excitations that are spectrally close to one another^5–7^. Furthermore, the background tails from the elastically scattered zero loss peak (ZLP) can extend well into the visible range, completely obscuring optical excitations and band-gaps in both the visible and infrared regimes^8–10^.

The energy resolution can be improved through monochromation, where the electron beam is dispersed by energy in real space, then a narrow band of the dispersed beam is selected by a slit and compressed back into the electron probe^11^. The technique has been a valued asset to electron microscopy, and previous generations of monochromated STEMs have yielded impressive results for many years^12–17^. However, with these instruments there has been a constant challenge to maintain spatial resolution and adequate signal in the monochromated probe, while still reaping the positive effects of monochromation. Recent significant breakthroughs in monochromated STEM have broken the previous records of energy resolution in EELS (<10 meV), while still maintaining a sub-nanometer electron probe^18,19^. These breakthroughs have significantly improved the capacity to analyze optical effects in the visible regime, opening up the infrared regime, and providing pathways to the analysis of vibrational modes in the STEM. For the new generation of monochromated STEM significant new results have already been reported^20–25^, but a systematic examination of the typical performance and effects of monochromator is still needed.

In this Letter, we examine the performance of Nion’s aberration-corrected high energy resolution monochromated EELS-STEM (HERMES^TM^) at Oak Ridge National Laboratory. EEL spectra are acquired at many different levels of monochromation, and the effects on energy resolution and background reduction in the infrared are investigated. The convolution between the ZLP and EEL signatures for vibrational modes were also studied as a function of monochromation. Finally, limits of this new generation of monochromated EELS are tested, in terms of both sensitivity to faint low-loss signals, and in terms of spatial resolution at high levels of monochromation.

## Discussion

**Figure 1:**
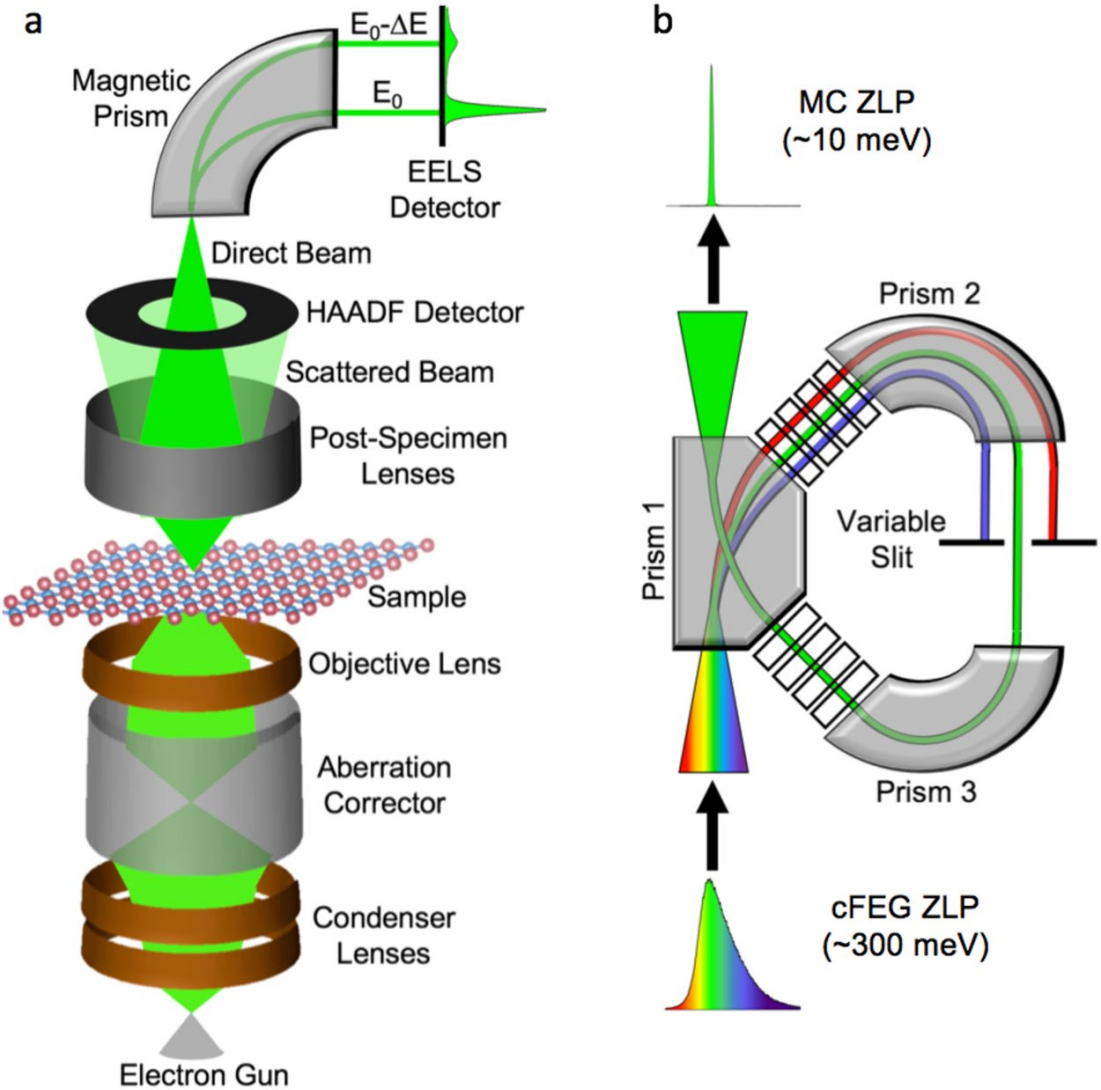
Electron energy loss spectroscopy and monochromation. (**a**) Schematic of electron energy-loss spectroscopy (EELS) experiment in a scanning transmission electron microscope (STEM). (**b**) Schematic of monochromation of electron beam (occurring between the electron gun and the condenser lenses).

Electron energy loss spectroscopy is a commonly employed technique for nanoscale analysis, due to its ability to associate spectroscopic data with an atomic-sized probe. The basic principle of the spectroscopy is that the electron beam transmits through a sample, where it loses energy by generating excitations, then the transmitted beam is dispersed by a magnetic prism to create a spectrum of the energy loss events that occurred in the sample. Figure 1a shows a schematic of STEM operation and the process that the electron beam goes through before it is collected in the EELS detector. After the gun, the magnetic fields in the various lenses and the aberration corrector only affect the shape of the beam, without changing the energy. Thus, the ultimate energy resolution of any EELS measurement is limited by the spread of the energies of the electrons coming out of the gun.

The full-width half-maximum (FWHM) of the ZLP is generally taken as the figure of merit for describing the energy resolution of an electron microscope. The ZLP is the EELS peak composed of electrons that are transmitted through the sample without losing energy. For cold field emission guns (cFEGs), such as the one used in this experiment, the FWHM of the ZLP is between 270–350 meV. Thermionic emission guns or Schottky guns have a ZLP with a larger FWHM, usually around 700 meV^11,26,27^. While these widths are small compared to the operating voltage of the STEM (usually between 60–300 keV), they set a limit for the energy resolution of EELS, and hinder the ability to distinguish between peaks separated by less than those values – hundreds of meV. Furthermore, for low-loss phenomena (bandgaps, surface plasmons, etc.) the excitation probability can be quite low, meaning that these signals might be lost in the tails of the ZLP, and so the FWHM of the ZLP is not as important a figure of merit as is the ZLP width at 1/1000-max or at 1/10000 max^7,8^. Thus, the tails of the ZLP, which are significant out well into the 1–2 eV range, can reduce the sensitivity of EELS to measure low-loss energy phenomena, or obscure them completely.

The FWHM of the ZLP, and especially the tails, can be reduced significantly via monochromation. Figure 1b shows a schematic of how the technique works; an electron beam is dispersed through a prism, spatially separating the electrons by energy, then a narrow band of energies is selected by a monochromating slit and recompressed into the electron beam. The Nion HERMES utilizes magnetic prisms (similar to those used in the EEL spectrometer) to disperse the electron beam. This differs from some other monochromated electron microscopes, which use Wien filters employing crossed electric and magnetic fields normal to the beam direction to perform the energy dispersion^28–30^.

## Results

### Levels of Monochromation

**Figure 2:**
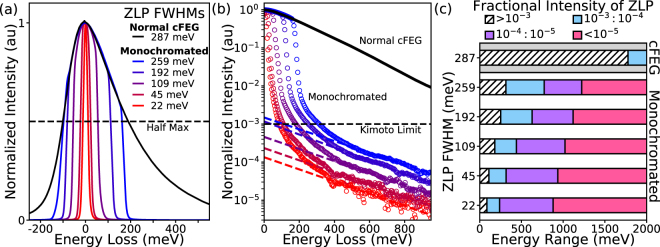
Effect of Monochromation on ZLP tails. (**a**) ZLPs of a non-monochromated cFEG gun (black), and the same gun with several different levels of monochromation (increasing from blue to red), showing increased energy resolution and reduced ZLP tails. (**b**) Log plots for the ZLPs shown in (a), demonstrating that the intensity of the ZLP tail is reduced by several orders of magnitude by monochromation. The dashed line marks where the intensity of ZLP tail falls below 10^−3^ (also known as the Kimoto limit), indicating that the background is small enough for effective EELS analysis. Dashed lines are power-law fits to the ZLP tails at higher energies. (**c**) The energy ranges at which the ZLP is at specific fractions of its total intensity. The hatched white bars show the ranges where the ZLP tails are above the the 10^−3^ threshold, while the other bars show where the ranges where the background is below 10^−3^ (blue), 10^−4^ (purple), and 10^−5^ (pink) of the ZLP maximum intensity.

Figure 2a shows the effect on the ZLP width and tails at different levels of monochromation. The non-monochromated ZLP is plotted in black, while the monochromated spectra are plotted in colors ranging from blue to red, corresponding to increasing levels of monochromation. The FWHM is measured for each spectrum, and in this example, monochromation improves the energy resolution from 287 meV for the non-monochromated beam down to 22 meV. However, the benefit of monochromation is not only in the improved resolution, it is in the reduced background from the removal of the ZLP tails. At a low level of monochromation (using a wide slit setting), the FWHM of the ZLP is not significantly improved from the normal cFEG FWHM, down to 259 meV from 287 meV, while the tails of the ZLP have been drastically reduced.

Figure 2b shows the normalized ZLPs from Fig. 2a on a logarithmic scale to better illustrate the impact of monochromation on the tails. In the cFEG spectrum, the ZLP tail is still at approximately 1% of its maximum intensity at an energy loss of 1 eV. This background is problematic, because it has been shown that EELS signatures are difficult to observe until the ZLP tail intensity has dropped below 10^−3^ of its maximum (known as the Kimoto limit)^8,10^. Increasing the monochromation results in ZLP tails that drop below the Kimoto limit at much lower energy losses, allowing features in the infrared regime to be resolved.

Figure 2c illustrates the energy loss ranges at which the non-monochromated and monochromated ZLP tails (shown in Fig. 2a,b) have different fractions of their maximum intensity. The top-most bar corresponds to the non-monochromated cFEG ZLP. The white hatched and blue bars represent the energy range where the ZLP tail intensity is either above or below the Kimoto limit, respectively. For the cFEG, the ZLP intensity does not drop below the 10^−3^ threshold until almost 2 eV (~620 nm wavelength). Such tails prohibit EELS analysis across the entirety of the infrared and much of the visible regime. For monochromated EELS, the background in the infrared is now below the limit, with the highest level of monochromation shown (22 meV FWHM) dropping below a fractional intensity of 10^−3^ at 86 meV (~15 μm wavelength). Furthermore, now both backgrounds in the infrared and visible regimes are not only below the Kimoto limit, they are orders of magnitude below the limit.

Figure 2c also shows the ranges where the ZLP tails are 10^−4^ (purple) and 10^−5^ (magenta) of their maximum intensity, with the 22 meV FWHM ZLP dropping below 10^−5^ at 879 meV (~1.5 μm wavelength), nearly a full eV less than the value at which the non-monochromated ZLP tails drop below the Kimoto limit (1776 meV). For the 10^−5^ energy range, the intensity values for each ZLP are extrapolated from a power-law fitting of the ZLP tails (dashed lines in Fig. 2b), while for the other intensity thresholds the signal-to-noise ratio (SNR) is high enough to measure them directly from the acquired data.

**Figure 3:**
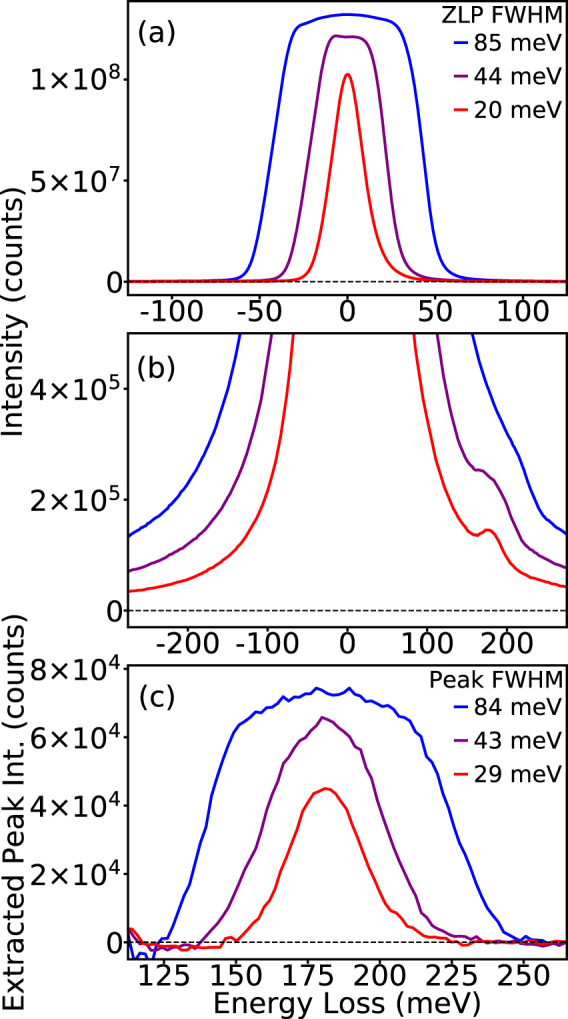
Effect of monochromation on low energy-loss signals (<500 meV). (**a**) ZLPs at different levels of monochromation with FWHMs of 85 meV (blue), 44 meV (purple), and 20 meV (blue). (**b**) The EEL spectra are taken from h-BN nanoflakes in aloof configuration. The h-BN phonon peak is observed in all three spectra with increasing sharpness as the monochromation increases. (**c**) Same as (**b**) with an expanded scale, after background subtraction. The FWHM of the phonon peaks correspond to the FWHMs of the ZLPs except for the 20 meV FWHM spectrum, which has broadened to 30 meV, indicating that the width of the h-BN phonon mode being observed possesses a comparable line-width.

The key benefit of this new generation of instruments is the accessibility of infrared excitations in the spectra, such as phonons, in a high-spatial-resolution electron microscope. In Fig. 3, the low-loss signature of hexagonal boron nitride (h-BN) is examined in an aloof spectroscopy configuration, where the beam is positioned in the vacuum near to the sample (~10 nm away). This spectroscopy configuration allows for delocalized excitations, like phonons, to be detected without incurring bulk losses or elastic broadening in the signal^23,31^. The EELS ZLP at three different levels of monochromation are shown in Fig. 3a. The ZLPs have FWHMs of 85 meV (blue), 44 meV (purple) and 20 meV (red).

The dominant phonon mode for h-BN occurs at 183 meV, and the EELS peak corresponding to the phonon is shown at each of the three levels of monochromation in Fig. 3b. In this example, the background from ZLP tails is not drastically different between the spectra, but the visibility of the phonon peak in the spectrum is significantly improved at the higher levels of monochromation. The reason for the enhanced clarity of the BN phonon in the highly monochromated spectra, is that the recorded EELS signal is effectively a convolution of the spectrum with the ZLP. To explore this effect, and to examine the linewidth and intensity of the phonon peaks, the background of the ZLP is fit with a power-law model and subtracted, as shown in Fig. 3c. It can be seen that the intensity and width of the phonon peaks correspond to the relative intensities and shapes of the ZLPs in Fig. 3a. The only significant disparity is the ZLP with the highest level of monochromation, which is broadened by 50%. The broadening indicates that at this level, the width of the phonon peak is comparable to the width of the ZLP and hence the convolution is no longer dominated by the ZLP width, and the genuine linewidth of the feature shows through.

### Background subtraction in the infrared

**Figure 4:**
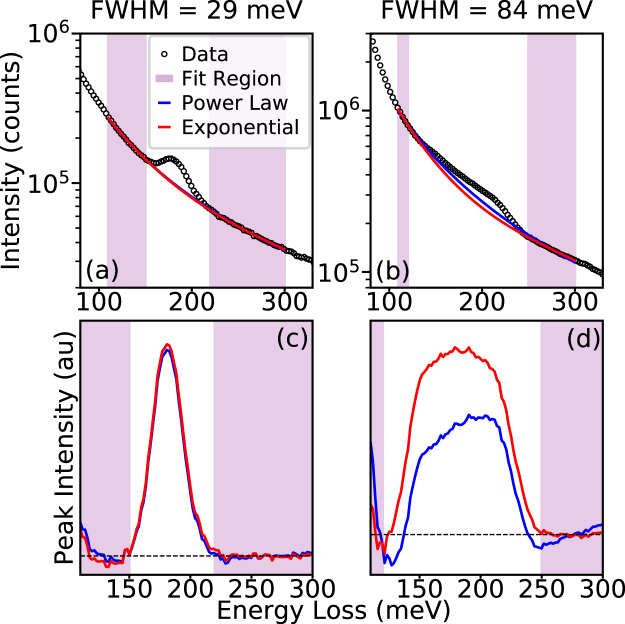
Fitting peaks on monochromated ZLP tails. (**a**,**b**) Log plots of monochromated BN phonon peaks from Fig. 3 with peak FWHMs of 29 meV (**a**) and 84 meV (**b**). In each the background has been fit with a power law (blue) and a third order exponential (red). In (**a**) the two fits are almost identical and overlap, while in (**b**) a difference can be observed. (**c,d**) The extracted BN phonon peaks from the different backgrounds shown in (**a**) and (**b**). With a small FWHM (**c**) there is not a significant difference between the two backgrounds, but for the larger FWHM (**d**) the third order exponential provides a much more accurate fit.

It is important to discuss the method by which the background is subtracted for the peaks in Fig. 3c. In EELS quantification, the standard background subtraction method is to fit a power law, $I(\triangle E)={A}_{0}\bullet {{\rm{\Delta }}E}^{-r}$, however there are many circumstances and regimes in which the power law background subtraction is not suitable, and different background fitting methods (such as exponentials or polynomials or combinations of different methods) are often more appropriate, especially near the ZLP^4,32–35^. In Fig. 4, the difference between power-laws high-order exponentials for background fitting in highly monochromated spectra.

Figure 4a shows the spectrum from Fig. 3b with the highest level of monochromation (red in Fig. 3b), while Fig. 4b shows the spectrum with the lowest (blue in Fig. 3b). Since the ZLP background in both spectra clearly possess a positive second derivative on the logarithmic scale, a standard exponential fit, $I({\rm{\Delta }}E)={e}^{-a\cdot {\rm{\Delta }}E-b}$, cannot accurately fit the background. By changing the exponent from a linear expression to a higher order polynomial,$\,I({\rm{\Delta }}E)={e}^{-a\cdot {\rm{\Delta }}{E}^{2}-b\cdot {\rm{\Delta }}E-c}$ or $I({\rm{\Delta }}E)={e}^{-a\cdot {\rm{\Delta }}{E}^{3}-b\cdot {\rm{\Delta }}{E}^{2}-c\cdot {\rm{\Delta }}E-d}$, the positive second derivative can be obtained. However, for a second order exponential to have a positive second derivative in log scale the coefficient of the second order term in the exponent would need to be positive, which would result in the expression diverging to infinity at high energies. Thus, a third order exponential is the lowest order exponential fit suitable for fitting the ZLP background in this energy regime. In Fig. 4a,b, the power law (blue) and a third order exponential (red) backgrounds are fit to energy ranges just before and after the BN phonon peaks and plotted against the original spectrum.

The results of the different types of background subtraction are shown in Fig. 4c,d. In Fig. 4c the extracted peaks of the highly monochromated BN phonon are shown. Here, because of the narrow linewidth both methods produce approximately the same fit and there is little benefit from using the exponential. However, in Fig. 4d, the extracted peaks from the weakly monochromated BN phonon show that the background subtraction method does make a significant difference. Here because the feature is broader there is more separation between the two fitting locations and the power law cannot be accurately fit for both, resulting in a non-physical extracted peak. The third order exponential provides a much better fit to the background on both sides of the peak as well as producing a symmetric BN phonon peak shape.

The improvement with the third-order polynomial is because several different factors contribute to the energy width of the monochromated electron probe. The natural energy distribution of the STEM-cFEG is determined by the Fowler-Nordheim equation,

1 $$I({\rm{\Delta }}E)\propto \frac{{e}^{{\rm{\Delta }}E/d}}{1+{e}^{{\rm{\Delta }}E/kT}}$$

where *d* is a constant determined by the Fermi-level, work function, and radius of the tip^36^. Even without monochromation it is difficult to accurately fit this highly asymmetric function with standard models like Gaussians, Lorentzians, and power-laws, but in the case of monochromation the Fowler-Nordheim distribution is further complicated by becoming convolved with a slit-induced cutoff. As a result, the more complex model fits the complex background more accurately in this spectral regime.

In Fig. 3c, as well as in the upcoming figures, a third order exponential is used to fit the background of the ZLP tail, as opposed to the power law. For a strong peak such as the one shown in Fig. 4a, the difference between the two subtraction methods is not likely to matter. However, for weaker peaks that are not so far above the noise level, accurate background fitting is required to avoid artifacts.

### Increased Sensitivity to Weak Signals

Another advantage of the increased sharpness of EELS excitations under high monochromation, beyond the increased precision and resolution, is that it also provides higher sensitivity to weak signals. However, if one acquires spectra with long dwell times to maximize the signal-to-noise ratio (SNR) of a peak that is much weaker than the ZLP, the detector of the EEL spectrometer can be saturated by the ZLP. This situation is problematic for several reasons. Firstly, on many instruments saturation can cause afterglow, coming from the scintillator in the spectrometer that can last hours. Additionally, there can be long-term radiation damage, which reduces the accuracy of EELS measurements and even the usable area on the camera. However, these afterglow effects are typically worse at lower temperatures, whereas in the prototype spectrometer used for these experiments the scintillator is optically coupled to the camera, allowing for the camera to be cooled but the scintillator to stay at room temperature. Secondly, even with a known dispersion and even without interference from scintillator afterglow, the relative energy loss can only be precisely calibrated by using the maximum of the ZLP as the true zero loss, and with the camera saturated the maximum of the ZLP is obscured.

**Figure 5:**
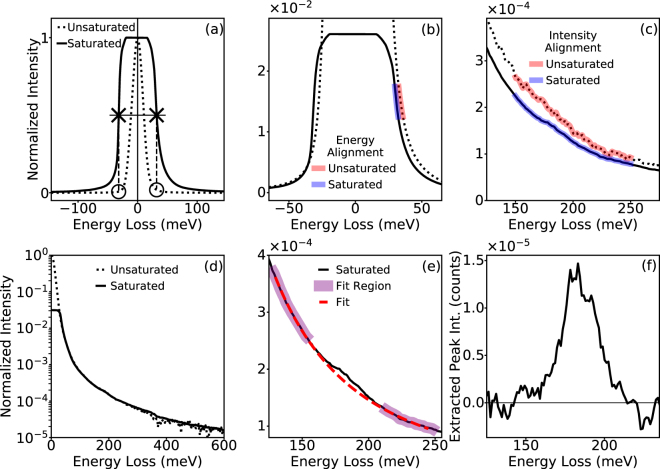
Extracting weak signals from saturated ZLPs. (**a**) ZLPs of EEL spectra taken from h-BN nanoflakes in aloof configuration, far away from the BN (~1 μm). One is acquired with long dwell times to maximize signal, but results in a saturated ZLP (solid line), the other is acquired with shorter dwell times to preserve the unsaturated ZLP (dashed). In order to calibrate and normalize the saturated spectrum it is aligned to the unsaturated ZLP. A rough alignment by centering the FWHM of the saturated peak on the true zero loss of the unsaturated peak, and then scaling the saturated spectrum by average intensity of the half maxes of the saturated spectrum (Xs) with the average intensities of the unsaturated spectrum at the same energy losses (Os). (**b**) Precise energy alignment is performed by taking a range of intensities from the unsaturated and saturated spectra (red and blue, respectively), and adjusting the energy loss axis of the saturated spectrum so the average energies in that range match. (**c**) Precise intensity alignment done similarly, by matching the average intensity in a specific energy range. (**d**) Result is a calibrated normalized spectrum with a saturated ZLP. (**e**) Background fitting shows observable BN phonon peak in saturated spectrum. (**f**) Extracted aloof BN phonon peak with intensity 5 orders of magnitude less than the ZLP maximum.

In order to calibrate and normalize saturated spectra they should be aligned to a spectrum where the ZLP is not saturated. In Fig. 5 the aloof excitation of the BN phonon is shown again, only this time the distance between the electron beam and the BN is increased from ~20 nm to ~1 μm. The delocalization of the BN phonon still allows for the mode to be excited by the electron beam, even at such a large distance, but with significantly reduced intensity, making high-SNR acquisitions critical for detecting the peak. Figure 5a shows two spectra, one where the ZLP is saturated (solid line) and one where it is not (unsaturated). The saturated spectrum is acquired by summing 10 separate spectra acquired with 10 second dwell times, while the unsaturated spectrum is summed over 100 spectra with 100 ms dwell times. The two spectra are acquired at the same beam position with the same beam conditions, but the saturation of the camera causes the ZLP to appear ‘clipped’.

To align the two spectra, we first find the energies of the ZLP half-maxima on both tails for the saturated spectrum. The half-way mark between the two energies is centered on the true zero loss of the unsaturated spectrum for a rough energy alignment, and then the entire saturated spectrum is scaled by the ratio between the average intensity of the saturated and unsaturated spectra at those energy values for a rough intensity alignment. Figure 4b shows that the rough alignment is not sufficient for accurate EELS measurements, so a more precise energy alignment is performed by finding the average energy across a selected intensity range in both spectra (red for unsaturated, and blue for saturated), and adjusting the energy loss axis of the saturated spectrum by the difference. The precise intensity alignment is performed similarly, by taking the average intensity over the same energy range from both spectra and adjusting the scaling factor by the difference. The result of this alignment is shown in Fig. 5d where it can be seen that now the ZLP tails of the saturated and unsaturated spectra match in both energy and intensity out to high energy losses.

With the saturated spectrum properly calibrated, the aloof BN phonon peak can now be extracted. Figure 5e shows the BN phonon peak of the saturated spectrum with a third order exponential background fit and the extracted peak is shown in Fig. 5f. Since the spectrum intensity has also been calibrated, it can be seen that the extracted is peak is ~10^−5^ of the maximum ZLP intensity, with a noise level (calculated from the background fitting regions) of ~10^−7^. The two orders of magnitude difference between the signal strength and the noise level illustrates how monochromation enhances the detectability of weak signals, through increased energy resolution and reduced ZLP tail intensity.

### Spatial Resolution

Finally, it is important to note that while the improved energy resolution is highly valuable, similar levels of energy resolution have been available for many years, as demonstrated by the pioneering work of Boersch in the 1960’s^12^. The importance of this new generation of monochromated STEMs is the ability to combine high energy resolution with high spatial resolution, while still maintaining enough probe current for simultaneous imaging and spectroscopy in practical timescales. Thus it is important to examine the achievable spatial resolution at varying levels of monochromation.

**Figure 6:**
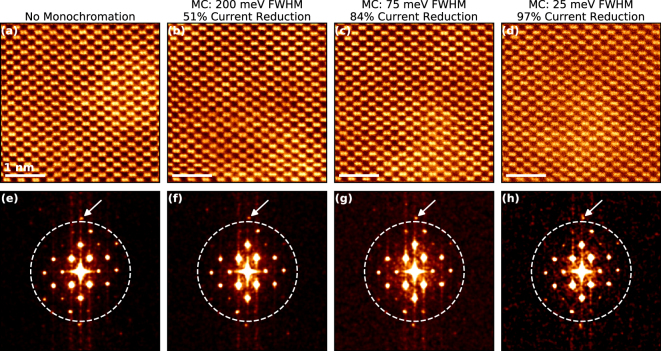
Monochromation and Spatial Resolution. (**a–d**) High angle annular dark field (HAADF) scanning transmission electron microscopy (STEM) images of the Si <110> zone axis at an accelerating voltage of 60 kV. (**e**,**f**) Shows the fast Fourier transform (FFT) of the HAADF images in (**a**–**d**) respectively. The dashed line in the FFT corresponds to a spatial resolution of 1 Å, Fourier points corresponding to smaller spacings are observed in all FFTs, indicating that sub-Å resolution is achieved on a probe with a ZLP FWHM of 25 meV.

Figure 6 shows atomic-resolution high angle annular dark field (HAADF) images of a Si <110> lattice at four different levels of monochromation, with the fast Fourier transforms (FFT)s of the images in the inset. Figure 6a shows the non-monochromated image. For an aberration corrected STEM at 60 kV the Si dumbbells, which have a separation of 1.36 Å in the <110> are resolvable, such features make good benchmarks for establishing for spatial resolution in imaging^37^. Figure 6b−d show HAADF-STEM images for monochromated probes with FWHMs of ~200 meV (b), ~75 meV (c), and ~25 meV (d). For all three levels of monochromation the Si <110> dumbbells are still resolved.

Furthermore, the level of spatial resolution can be partially measured through the FFTs. Figure 6e–h show the FFTs of the HAADF images in Fig. 6a–d. In each FFT there is a dashed circle which corresponds to atomic spacings of less than 1 Å in the real-space image. Fourier spots observed outside of the circle in the FFT, strongly suggest that sub-Å resolution is present in the image. At the very top of the dashed circle, a bright Fourier spot that corresponds to a spacing of 0.96 Å is observed in all four images^38^, meaning that sub-Å resolution information transfer can still be achieved, even with a highly monochromated beam.

In the conditions used in our experiments we did not find an improvement in the spatial resolution at 60 kV after monochromation. However, in lower-voltage (S)TEM (<60 kV) one of the predominant limiting factors in the spatial resolution of the electron probe is chromatic aberration. The focal spread will depend on the product of the chromatic aberration coefficient of the objective lens (Cc) and the energy spread. Under the conditions used here, these chromatic effects primarily increase the probe tails. Thus, we might expect monochromation to produce some improvement in the image SNR. While for our system the chromatic blurring is reduced via monochromation, the corresponding reduction of the total signal decreases the SNR too much for us to detect an improvement in <110> Si. The total reduction in beam current due to monochromation is shown at the top of each image, and for the highly monochromated case it can be seen that the total beam-current is decreased by nearly two orders of magnitude. However, others have observed monochromation to improve spatial resolution in TEM^39^, as well as STEM at different accelerating voltages^19^.

## Conclusion

In conclusion, monochromation provides significant advantages for the spectroscopic analysis of ultralow-loss phenomena. By removing the broad probe tails of the ZLP, different levels of monochromation can improve various aspects of EELS analysis in a variety of different ways. Here, we have illustrated how these new monochromated microscopes can efficiently balance the required total signal with the desired energy resolution, background levels, excitation sensitivity, and imaging capability to optimize nanoscale experiments. The reduced backgrounds and increased sensitivity due to monochromation have allowed for rigorous optical EELS experiments to access excitations and systems where the signal had previously been too weak, such as extremely small (<5 nm) nanocrystals^21^, excitons at 2D material interfaces^40^, and mid-gap defect states^41^. Furthermore, we have demonstrated that even in highly monochromated beams, the spatial resolution of the electron microscope is not compromised and still reaches to the sub-Å regime. While bulk vibrational modes are delocalized to an extent where sub-Å resolution is not a significant advantage, momentum-resolved EELS can be used to access points in the dispersion curve where the phonons are highly localized^22^. The simultaneous spatial- and spectral-resolution of monochromated STEM-EELS has been used to study nanoscale confinement effects on structures like carbon nanotubes^25^ and MgO nanocubes^24,42^. This new echelon of monochromated EELS is still in its infancy, and as experimentalist we will need to learn how to fully take advantage of these new technological advances, in order to deliver on the promise of advanced understanding of atomic and nanoscale phenomena across materials science.

## Methods

All experiments in this manuscript were performed on the Nion’s aberration-corrected high energy resolution monochromated EELS-STEM (HERMES^TM^) at Oak Ridge National Laboratory operated at 60 kV accelerating voltage. The HERMES at Oak Ridge is equipped with a prototype Nion spectrometer possessing a Hamamatsu ORCA high-speed cMOS detector.

The EEL spectra shown in Figs 2–5 were all acquired with a 1 mm EELS aperture corresponding to a collection angle of 13 mrad, a probe with a convergence angle of 15 mrad, and a beam current of ~300 pA. All shown EEL spectra were produced by taking multiple acquisitions then using sub-pixel alignment to create a single summed spectrum, with the goal of minimizing the effect of tip-noise on the EELS. For Figs 2, and 3a the shown spectrum is summed over 100 acquisitions with a 100 ms dwell time to leave the ZLP unsaturated. The remainder of spectra in Fig. 3, as well as all of those in Figs 4 and 5, were the summed over 10 acquisitions with a 10 s dwell time to maximize SNR, and aligned to 100 acquistion/100 ms dwell time spectra to calibrate and normalize.

The probe used for the imaging in Fig. 6 had a convergence angle of 30 mrad and a beam current of 20 pA. The HAADF detector had a nominal inner collection angle of 90 mrad and an outer collection angle of 200 mrad. All images shown were 1024 × 1024 pixels across a 4 nm FOV with a 32 μs dwell time, the beam current reduction is calculated by integrating the total intensity in the ZLP in the vacuum away from the sample, and then comparing to the total ZLP intensity in the unmonochromated probe. The FFTs and images in Fig. 6 have a Gaussian blur (σ = 1) to reduce noise and show the atomic resolution more clearly.

### Data availability

The datasets generated during and/or analysed during the current study are available from the corresponding author on reasonable request.
